# Analysis of Placental Arteriovenous Formation Reveals New Insights Into Embryos With Congenital Heart Defects

**DOI:** 10.3389/fgene.2021.806136

**Published:** 2022-01-19

**Authors:** Jacinta I. Kalisch-Smith, Emily C. Morris, Mary A. A. Strevens, Andia N. Redpath, Kostantinos Klaourakis, Dorota Szumska, Jennifer E. Outhwaite, Xin Sun, Joaquim Miguel Vieira, Nicola Smart, Sarah De Val, Paul R. Riley, Duncan B. Sparrow

**Affiliations:** ^1^ BHF Centre for Research Excellence, Department of Physiology, Anatomy, and Genetics, University of Oxford, Oxford, United Kingdom; ^2^ Nuffield Department of Medicine, Ludvig Institute for Cancer Research Ltd., University of Oxford, Oxford, United Kingdom; ^3^ School of Biomedical Sciences, University of Queensland, Brisbane, QLD, Australia

**Keywords:** placenta, endothelial, labyrinth, allantois, iron deficiency, congenital heart defects

## Abstract

The placental vasculature provides the developing embryo with a circulation to deliver nutrients and dispose of waste products. However, in the mouse, the vascular components of the chorio-allantoic placenta have been largely unexplored due to a lack of well-validated molecular markers. This is required to study how these blood vessels form in development and how they are impacted by embryonic or maternal defects. Here, we employed marker analysis to characterize the arterial/arteriole and venous/venule endothelial cells (ECs) during normal mouse placental development. We reveal that placental ECs are potentially unique compared with their embryonic counterparts. We assessed embryonic markers of arterial ECs, venous ECs, and their capillary counterparts—arteriole and venule ECs. Major findings were that the arterial tree exclusively expressed *Dll4*, and venous vascular tree could be distinguished from the arterial tree by Endomucin (EMCN) expression levels. The relationship between the placenta and developing heart is particularly interesting. These two organs form at the same stages of embryogenesis and are well known to affect each other’s growth trajectories. However, although there are many mouse models of heart defects, these are not routinely assessed for placental defects. Using these new placental vascular markers, we reveal that mouse embryos from one model of heart defects, caused by maternal iron deficiency, also have defects in the formation of the placental arterial, but not the venous, vascular tree. Defects to the embryonic cardiovascular system can therefore have a significant impact on blood flow delivery and expansion of the placental arterial tree.

## Introduction

Delivery of oxygen and nutrients, and the disposal of waste products are both essential for an embryo to grow and develop. In mammals, these functions are provided by the placenta. In this organ, the maternal and embryonic bloodstreams are juxtaposed, providing an interface for nutrient and waste exchange. In the mouse, the chorio-allantoic labyrinth vasculature connects through the umbilical cord to the embryonic blood circulation. The labyrinth is made up of arteries and veins that differentiate from the allantoic mesenchyme, an extra-embryonic tissue that arises from the end of the primitive streak during gastrulation. Morphogenesis of this vasculature begins at embryonic day (E)7.5, whereupon several stem cell populations feed into the allantoic bud and transform into a branched endothelial cell (EC) network expressing CD31 (Drake and Fleming, 2000; Downs and Rodriguez, 2019). These allantoic ECs penetrate the base of the placental chorion, branch, and form the one EC layer that makes up the interhemal membrane (Simmons et al., 2008; Cross et al., 2003). Branching continues to make two adjoining vascular trees, one arterial and one venous, which both connect to the umbilical cord. The umbilical artery is remodeled at E8.5 from the allantoic vascular plexus into a centralized vessel; however, virtually nothing is known about how and when the umbilical vein is formed. Beyond these morphological events, very little is understood of the cellular and molecular processes driving placental vascular development. This is because w*e currently have no molecular way to identify and define the cells and structural components of the placental vascular tree.*

It is important to understand how the placental arterial and venous vasculature forms as they can be impacted by environmental perturbations and genetic knockouts, particularly those which impact blood flow from the heart (Detmar et al., 2008; Bainbridge et al., 2012; Withington et al., 2006; Adamson et al., 2002). Experiments mimicking reduced blood flow from the placenta cause a variety of heart defects (Midgett et al., 2017). The heart must beat against the resistance of the placental vascular bed (Thornburg et al., 2010), and therefore, changes to placental development and blood flow are likely to impact the forming embryonic heart. Similarly, perturbations to either the placenta or the heart can result in impacts to both (Perez-Garcia et al., 2018; Shaut et al., 2008; Araujo Júnior et al., 2016). This is particularly important as congenital heart disease (CHD) impacts almost 1% of births worldwide (Liu et al., 2019) and can be due to either genetic defects (Alankarage et al., 2018) or perturbation of the maternal environment (Kalisch Smith et al., 2019).

Historically, the placental vascular trees have been identified by using plastic vascular casts or microcomputed tomography imaging (Adamson et al., 2002; Rennie et al., 2017). However, analysis of the placental arterial and venous EC vasculature has stalled in recent years due to a lack of identifying markers. Thus, there is little known of how they differentiate, the genetic programs they use prior to the onset of flow and during maturation, and whether they are molecularly distinct from other vascular beds in the embryo. Two subsets of ECs have been identified at E10.5. Candidate markers *Vegfa* and *Vegfc* have been proposed as arterial and venous markers, respectively (Outhwaite et al., 2019). The *Apelin receptor Aplnr (Apj)* may also be expressed solely in veins (Outhwaite et al., 2019). In the embryo, these are well-defined markers of arterial and venous specification and so may be acting in a similar manner in the placenta. The placental endothelium may also express other genes similar to the systemic embryonic endothelium, such as vein EC-specific *Eph receptor B4* (*EphB4*) and artery EC-specific *Ephrin B2* (*Efnb2*). These are expressed at E9.5 in the umbilical vein and artery, respectively (Wang et al., 1998), which connect to placental arterial and venous vessels, and are also allantoic-derived. However, we do not know whether these umbilical markers are also expressed on EC vessels once they enter the placenta and branch to create capillaries.

Little is known about venous differentiation in the placenta. Recent single-cell transcriptomic analysis of labyrinthine cells identified a unique cell type cluster containing both allantoic mesenchymal cells and ECs, and an additional cluster marked by *Podoplanin* (*Pdpn*) expression (Marsh and Blelloch, 2020). However, this study did not distinguish between arterial and venous ECs nor elaborate on the nature of the *Pdpn*-enriched cluster. A more recent single-cell transcriptomic analysis of the E10-E12 placenta (Liang et al., 2021) identified separate clusters for arterial and venous cells, expressing *Gja4 (Cx37)* and *Nr2f2 (CoupTF-II)*, respectively. However, these expression profiles are yet to be confirmed *in situ*. In conclusion, the exact nature of the molecular pathways underlying arterio-venous development in the placenta is unclear. We hypothesized that these pathways are similar to the well-characterized mechanisms of arterio-venous development in the embryo. To test this hypothesis, we investigated the expression of well-described embryonic vascular gene markers in E10.5 to E15.5 mouse placentas. Here, we find that five vascular cell types of the placental labyrinth: arterial ECs, venous ECs, their capillary equivalents (arterial and venule ECs), and pericytes express distinct markers to their embryonic counterparts. Unexpectedly, we also find expression of markers of the embryonic lymphatic vasculature in all placental ECs (LYVE1+, PDPN+) and in placental mesenchymal cells (PDPN+). Finally, we used these new markers of placental arterial and venous ECs to investigate the placental phenotype of a mouse model of environmentally caused CHD (Kalisch-Smith et al., 2021).

## Methods

### Animal Housing and Diet Modification

All animal experiments were compliant with the UK Animals (Scientific Procedures) Act 7391986, approved by the University of Oxford animal welfare review board and the Home Office (project license PB01E1FB3). Mice were housed in an SPF facility with a 12-h:12-h light/dark cycle, at 19°C–23°C, 55 ± 10% humidity, with free access to food and water. Bedding was changed fortnightly, and animals were assessed daily for welfare. C57BL/6J mice (Charles River United Kingdom) were fed with standard chow TD.08713 (control) or TD.99397 (iron deficient) feed (Envigo, Belton, United Kingdom). The following genetically modified mouse strains were used; *Lyve1*-Cre (JAX, 012601, *Lyve1* ^*tm1.1(EGFP/cre)Cys*^ *,* C57BL/6), R26R-tdTomato Ai14 C57BL/6J (Madisen et al., 2009), and 129X1/SvJ *Cx40*:GFP (*Gja5* ^*tm1Lumi*^) (Miquerol et al., 2004). Pregnant mice were sacrificed humanely, and placentas were dissected in 1× Hanks’ balanced salt solution +10 mM EDTA. E9.5 embryos were counted for somite number. Placentas were weighed and fixed in 4% paraformaldehyde overnight. The following day, placentas were bisected with a double-edged blade, with half allocated for paraffin embedding and half for cryosectioning. Both were embedded cut face down. HREM placentae remained whole.

### High-Resolution Episcopic Microscopy Assessing Heart and Placental Morphology

Somite-matched placentas at E9.5 were fixed in 4% paraformaldehyde and processed in a methanol series (10%, 20%, 30%, 40%, 50%, 60%, 70%, 80%, 90%, 95%, and 100%) for 1-h washes each. Samples were incubated in 50:50 mixture of methanol and JB4 resin (Polysciences, 00226-1, GMBH, Germany) overnight. Samples were incubated in JB4 resin for 1 h and transferred to fresh resin for incubation overnight. E15.5 embryos were fixed overnight in 4% paraformaldehyde at 4°C and were dissected for heart–lung complexes. These samples used the same conditions as above, except the final JB4 incubation, which was for 3 days. Samples were embedded individually in JB4 according to the manufacturer’s instructions. Samples were cut (2-µm E9.5 placenta, 3-µm E15.5 heart–lung complex) on an optical HREM (OHREM) microscope (Indigo scientific) with images taken using a Jenoptik Gryphax camera. Image stacks were processed into cubic data and reduced to either 10% or 50% for 3D modeling using the Amira software package 2019.4 (Thermo Fisher Scientific). Grayscale images were imported into MD DICOM viewer version 9.0.2 (Pixmeo), or Horos 3.3.6 (https://horosproject.org) in a 50% stack, inverted to black–white, and rendered into 3D.

### Immunohistochemistry, Imaging, and Placental Quantification

IHC on paraffin sections was performed as previously described (Shi et al., 2016). Paraffin-embedded tissue was cut at 7 µm. ID samples at E12.5 and E15.5 were used for stereology as described by Coan et al. (2004). E12.5 tissue was sampled at 1:25, whereas E15.5 tissue was sampled at 1:50 sections. Slides were dewaxed with xylene and rehydrated with an ethanol series prior to antigen retrieval in either TE (pH 8.5) or citrate buffer for 20 min, with 20-min cool down. Slides were processed using a Shandon Sequenza^®^ Immunostaining Center (Thermo 827 Fisher Scientific). Slides were blocked in 2.5% donkey/goat serum in PBST for 45 min at RT and then in Sudan black for 20 min to removed autofluorescence. Slides were washed in 3× PBST prior to incubation in primary antibody (in 2.5% block in PBST) overnight at 4°C see Table 1. Slides were washed 1× PBST prior to incubation in secondary antibody (in 2.5% block in PBST) for 1 h. For fluorescent slides, this included TO-PRO®-3 Iodide nuclear dye (T3605, Life Technologies 1:10,000). Slides were washed 3× PBST prior to mounting with anti-fading PVA/Moviol-DABCO (Sigma) medium. Fluorescent slides were imaged using an Olympus FV3000 confocal microscope using an Olympus UPLSAPO NA0.4 10× objective. Individual images were captured at 1,024 × 1,024 pixel resolution.

**TABLE 1:** Antibodies and optimised conditions for immunohistochemistry.

Antibody	Species/Conjugation	Clonality	Dilution	Company	Product code	Antigen retrieval/Cryosections
ACTA2/a-SMA	Rabbit	Polyclonal	1:200	Abcam	ab5694	CRYO
BGAL	Chicken	Polyclonal	1:200	Abcam	ab9361	CRYO
CD105/ENDOGLIN	Goat	Polyclonal	1:25	R&D Systems	AF1320	CRYO
CD31	Armenian Hamster	Monoclonal	1:100	Abcam	ab119341	CRYO
ENDOMUCIN	Rat	Monoclonal	1:50	Santa Cruz	sc53941	CRYO
Isolectin B4	Biotin	—	1:200	Sigma	L2140	TE
Isolectin B4	Peroxidase conjugate	—	1:100	Sigma	L5391	TE or Citrate
LYVE1	Rabbit	Polyclonal	1:100	Angbio	11–034	CRYO
CSPG4/NG2	Rabbit	Polyclonal	1:100	Millipore	AB5320	Citrate
NOTCH1	Rabbit	Polyclonal	1:100	Abcam	Ab8935	CRYO
NRP1	Rabbit	Monoclonal	1:50	Abcam	ab81321	CRYO
NRP2	Rabbit	Monoclonal	1:50	Cell Signaling Tech	D39A5	CRYO
PODOPLANIN	Hamster	Monoclonal	1:100	Fitzgerald	10R-P155a	TE, Citrate or CRYO
MYH11/SM-MHC	Rabbit	Polyclonal	1:100	Abcam	Ab125884	CRYO

For isolectin B4 staining (ILB4), slides underwent TE antigen retrieval, washed in PBS for 5 min, and blocked for endogenous peroxidases using 0.9% H_2_O_2_ in MQ H_2_O for 10 min. Slides were then incubated in 0.1% triton with 0.1 mM ions (MgCl_2_, CaCl_2_, and MnCl_2_) for 10 min prior to incubation with ILB4 (derived from *Bandeiraea simplicifolia*) in PBS for 2 h at RT. Slides placed in DAB substrate (Vector peroxidase substrate kit, SK-4100) for color reaction. This reaction was stopped by washing in Milli-Q water. Following, slides were counterstained with hematoxylin and mounted in DPX medium. For light microscopy, whole placental sections were imaged at ×1.25 (objective) magnification using an Olympus SZX7 Compact Stereo Microscope (E12.5 and E15.5), and three fields of view of the labyrinth (E15.5) were imaged using a Nikon Eclipse Ci microscope at ×40 (objective) magnification. Placental compartments were then estimated using the Cavalieri principle, as described by Coan et al. (2004) and analyzed in ImageJ (NIH). A 100- and 200-μm^2^ grid was respectively superimposed onto each field of the ×1.25 and ×40 sections, and the number of points falling onto each placental compartment was counted, blind to treatment group. At E12.5, total, decidua, junctional zone, and labyrinth were estimated. At E15.5, fetal and maternal blood spaces were estimated in addition to these compartments.

### Identification of Allantois-Derived ECs

Pan-EC markers ILB4 and CD31 were used as a reference standard and positive control for each gene/protein marker investigated. Negative staining was based on background staining in various fluorescent channels. The umbilical artery and vein were localized to the base of the labyrinth, and their EC expression profiles matched those to the embryo. Localization of placental arteriole and venular capillaries were based on vascular cast studies performed previously (Adamson et al., 2002; Withington et al., 2006).

Counting of total and venous (EMCN^hi^) ECs and associated vessels was performed using AngioTool (Zudaire et al., 2011). Image stacks (*n =* 4) created an average intensity projection. CD31 images were used for total counts, whereas EMCN images were thresholded and cropped to only show venous positive vessels.

### RNAscope

RNAscope Multiplex Fluorescent v.2 assay (ACD) was performed on 12-µm cryosections according to the manufacturer’s instructions with minor modifications (previously described by Lupu et al., 2020). Slides were boiled for 8 min. Protease Plus digestion was performed for 30 min at RT. Probes were optimized for hybridization at 40°C. TSA plus fluorophores (Akoya Biosciences) used are Cy3 (1:1,500) and Cy5 (1:1,500). Slides were counterstained with DAPI. The following catalog probes were used: *Cd31* (#316721-C3), *Aplnr* (*Apj*, #436171), and *Dll4* (319,971). The 3-plex negative probe against dapB was used as a negative control (#320878). Negative control staining for mRNA showed background staining for the Cy3 channel and no staining for Cy5 (Supplementary Figure S2H). Assays were run in duplicate. Confocal imaging was performed as outlined above, except 4× Z stacks were taken. Average intensity projections were used as representative images.

### Statistical Analyses

All statistical analyses were performed with Prism 8.4.2 (GraphPad Software). Data were tested for normal distribution by Shapiro–Wilk tests. For data with two groups, normally distributed samples were analyzed by two-tailed Student’s t-test. Non-normally distributed samples were tested using a two-tailed Mann–Whitney test. For >2 groups, a parametric one-way ANOVA or non-parametric Kruskal–Wallis test was used. Statistical significance was set at <0.05. Data are presented as mean ± standard deviation (SD).

## Results

### Investigation of Allantoic-Derived Arterial and Venous Marker Expression in the Placenta

Prior to investigation of CHD and potential placental phenotypes, we first wanted to classify subsets of allantois-derived placental labyrinth cells. We profiled common EC markers to embryonic blood vessels previously investigated by Chong et al. (2011), including arterial ECs and venous ECs to determine whether placental blood vessels are patterned in the same manner as embryonic blood vessels. Markers for analysis were chosen for their roles in arteriovenous or lymphatic differentiation, response to blood flow, or cell localization and knockout phenotypes. We focused our analysis on different types of structures, which transport blood through the placenta, i.e., from the umbilical circulation (systemic artery/vein), via the biggest vessels entering the labyrinth, through to the arterial stems (primary branches of vascular tree) and to the terminal branches of capillaries (arterioles and venules). The E12.5–E15.5 time points were used because, by these stages, the placenta vascular structure has developed, and terminal EC differentiation has begun. Expression profiles were compared to the pan-EC markers CD31 and ILB4.

**FIGURE 1:**
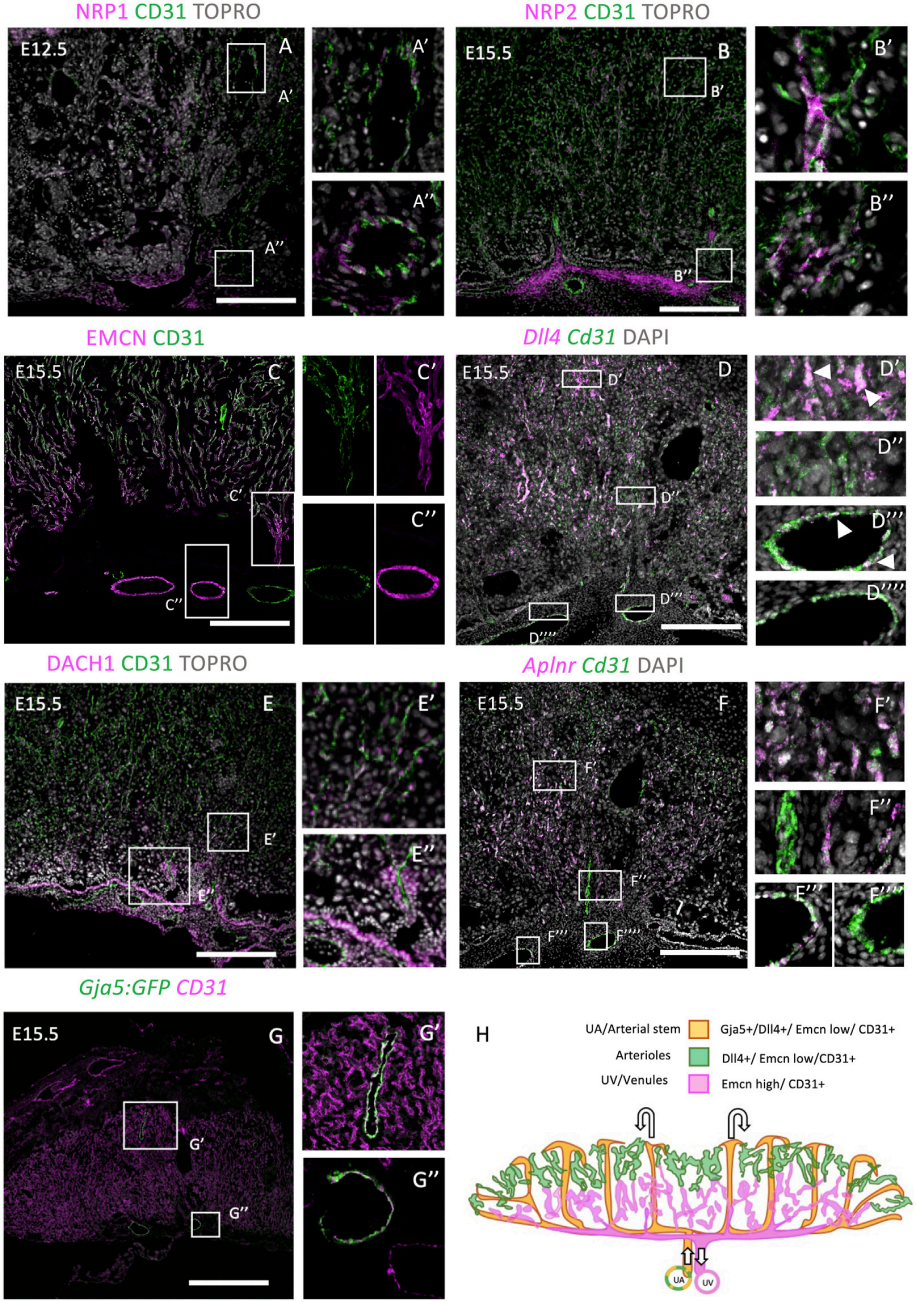
Investigation of embryonic arterial and venous genes during mouse placentation. E12.5 or E15.5 placentae were stained with embryonic arterial-specific genes (NRP1, DACH1, *Dll4*, and GJA5:GFP in A, D, E, and G), and embryonic venous-specific genes [NRP2, Endomucin (EMCN), and *Aplnr* in B, C, and F]. IHC was performed in **(A–C, E, F)**, whereas RNAscope was performed in **(F)**. The boxes outline the areas shown at higher magnification in **[A–G (‘)]**. **(H)** Diagrammatic representation of arterioles (green), arterial stems/uterine artery (orange), and venous/uterine vein (magenta) genes within the E15.5 placenta. Diagram was created by tracing cell markers and structures from placental sections. Scale bars (white) = 200 um.

Firstly, we investigated two neuropilin isoforms: NRP1 and NRP2. In the embryo, these are specific to arterial and venous ECs, respectively (Chong et al., 2011), and have roles in EC sprouting and lymphogenesis (Xu et al., 2010). For example, NRP1 can form a mechanosensory complex with PLEXIND1 and VEGFR2 in ECs (Mehta et al., 2020) or can bind other ligands including Semaphorins and VEGF164 to promote vascular development (Vieria et al., 2007). Remarkably, the placental vasculature showed a different expression pattern to embryonic ECs. NRP1 was found in large arterial vessels at E12.5 (Figure 1AA’,A’’), but not at E15.5 (Supplementary Figure S1B). In the embryo, this expression is maintained in arteries at this stage of embryogenesis (Erskine et al., 2017). NRP2 was present in a subset of CD31-positive ECs throughout the placenta at E15.5 and, sporadically, at E12.5 throughout the placenta (Figure 1BB’,B’’, Supplementary Figure S1C). This suggests that NRP2+ cells are not restricted to either arterial or venous ECs. Finally, both NRP1 and NRP2 were expressed in the allantoic mesenchyme.

In the embryo, Endomucin (EMCN) is restricted to capillaries and venous ECs from E15.5 in development and is involved in angiogenesis (Brachtendorf et al., 2001; Park-Windhol et al., 2017). By contrast, embryonic arterioles do not show EMCN staining (Brachtendorf et al., 2001; Cavallero et al., 2015). In the placenta, EMCN showed not only strong expression in veins and venules but also mild expression in arterioles at E12.5 and E15.5, respectively (Figure 1C, Supplementary Figure S2G). At both time points, high expression can be seen in collecting vessel ECs at the base of the labyrinth (Supplementary Figure S2G). Later, at E15.5, EMCN showed higher expression in venules within the lower portion of the labyrinth, compared with arterioles within the upper portion. Delta-like ligand 4 (*Dll4*), a Notch ligand, is a robust embryonic arterial EC marker from E8.0 (Chong et al., 2011) and is also present in the umbilical and vitelline arterial ECs and some placental vessel ECs at E9.5 (Duarte, 2004). *Dll4* was of particular interest as it is involved in the arterial specification cascade through Notch signaling, and therefore, its expression begins prior to flow (Chong et al., 2011). In addition, *Dll4* null mice show embryonic lethality by E10.5, with aberrant vessel formation and regression of the placental vasculature (Arora and Papaioannou, 2012; Duarte, 2004; Gale et al., 2004), suggesting that it plays a role in placental vascular formation. In the placenta, *Dll4* mRNA was more abundantly expressed in the umbilical arterial ECs than the vein ECs (Figure 1DD’’’ vs. D’’’’, magnified image in Supplementary Figure S1D) and detected in arteriole capillaries (see Figure 1DD’, D’’), colocalizing with *Cd31* mRNA. This agrees with the embryonic expression outlined above. DACH1 is a transcription factor that has been previously shown to promote EC migration and coronary artery growth, and its expression is stimulated by shear stress (Chang et al., 2017). More recently, DACH1 has been implicated in pre-artery specification and differentiation of arterial ECs (Raftrey et al., 2020, pre-print). In the placenta at E15.5, DACH1 showed strong expression in the ECs of collecting vessels (Figure 1EE’’, Supplementary Figure S2G) and nuclear expression in capillaries extending toward the junctional zone (Figure 1E’). Its expression is therefore similar to the embryo. *Apelin receptor (Aplnr)* is upregulated by VEGF signaling at E9.5 and facilitates vessel sprouting and branching (Freyer et al., 2017). It is restricted to one vessel, leading into the placenta (Freyer et al., 2017; Outhwaite et al., 2019). *Aplnr* is a venous marker in the retina (Saint-Geniez et al., 2002), cardinal vein (Chong et al., 2011), and the sinus venosus, a progenitor to the coronary vessels (Sharma et al., 2017). However, it can also be expressed by capillaries in the lung (Gillich et al., 2020), and it is also specific to allantoic ECs (Ho et al., 2017). *Aplnr* null mice exhibit vascular defects in the embryo and embryonic death from E10.5 (Kang et al., 2013). In the placenta, *Aplnr* mRNA puncta were highly localized to the umbilical vein ECs but not to the umbilical artery ECs or central arterial stems (Figure 1F). Similar to the patterns of EMCN expression, *Aplnr* was more highly expressed in basal venous ECs but was also present in arteriole ECs. This placental expression profile is therefore similar to other organs in the embryo. *Gja5* (also known as *Cx40*), an embryonic arterial marker, is a gap junction protein, and its expression is maintained by flow (Chong et al., 2011). Interestingly, in the placenta, *Gja5* showed expression in only arterial EC stems (Figure 1GG’) and umbilical artery ECs (G″). See Figure 1H for diagrammatic summary of expression profiles. Taken together, this analysis shows that the placenta only mimics a subset of embryonic expression patterns and therefore is a potentially unique endothelial organ bed.

To further understand labyrinth morphogenesis, we conducted further analysis of key vascular genes in the placenta at earlier developmental time points. CD105 (Endoglin) is an EC-expressed TGFβ co-receptor, which is required for angiogenesis, with null embryos showing perturbed arteriovenous formation and lethality at E10.5 (Li, 1999). Similar to the embryo, CD105 showed strong pan-EC expression at E12.5 (Supplementary Figure S2A). Actin alpha 2 (ACTA2, also known as αSMA) was found in labyrinthine and allantoic mesenchymal cells at E10.5 (Supplementary Figure S2B) and in a central artery projecting to the upper portion of the labyrinth. This expression pattern remained the same at E12.5 (Supplementary Figure S2C). *Notch1* expression has been previously identified in the embryo in arterial ECs and shows expression by E8.25 (Chong et al., 2011). In the placenta, NOTCH1 expression was primarily expressed in the smooth muscle of the umbilical artery at E15.5 (Supplementary Figure S2D), whereas MYH11 (SM-MHC, smooth muscle) was expressed highly throughout the allantoic mesenchyme, collecting vessels, and surrounding arterioles (Supplementary Figure S2E). In summary, we have found gene markers to identify placental ECs for arteries (*Gja*5+, *Dll4*+), arterioles (*Dll4*+), and veins and venules (EMCN^high^).

### Common Lymphatic Genes Are Expressed in the Placenta

Single-cell RNA approaches have identified that several lymphatic genes are expressed in the placenta (Marsh and Blelloch, 2020; Liang et al., 2021). This prompted us to question whether there may be a lymphatic network within the chorio-allantoic placenta. To date, lymphatic markers have only been investigated on the maternal side of the placenta (Pawlak et al., 2019) and found to be absent in the term human placenta (Becker et al., 2020). We first screened common lymphatic proteins (PDPN, LYVE1, and PROX1) for expression in the mid-late gestation placenta. The mucin-type protein podoplanin (PDPN) has been previously associated with epithelial–mesenchymal transition (EMT) and is expressed in lymphatic endothelium and epicardial cells (Thiery, 2002). In the placenta, PDPN was expressed in basal labyrinth cells (ILB4−) at E10.5, with continuing cells leading into the midline becoming ILB4+ ECs (Supplementary Figures S2B, S3A). Similar expression patterns were shown at E12.5 (Supplementary Figure S2A). By E14.5/E15.5, a subset of these PDPN+ cells was also ILB4+ and was seen in major vessels extending toward the junctional zone, small crypts near the base of the labyrinth, and in allantoic mesenchyme surrounding embryonic blood vessels (Supplementary Figures S3B,CC’’’). Co-staining showed that PDPN+ cells were adjacent to both EMCN+ blood vessels and CSPG4 (NG2)+ pericytes (Supplementary Figures S3D,E). These placental PDPN+ cells could be therefore a subset of mesenchymal and blood vessels undergoing EMT, as they are dispersed throughout the labyrinth. See diagrammatic representations for expression patterns at E10.5 (Supplementary Figure S3F) and E14.5 (Supplementary Figure S3G).

Lymphatic vessel endothelial hyaluronan receptor-1 (LYVE1) is expressed in embryonic blood vessels, lymphatic vessels, and the lymph node, among other organs (Gordon et al., 2008). *Lyve1* is reported to be expressed in labyrinthine ECs, albeit only at E11.5/E12.5 (Shaut et al., 2008). To investigate further, we used the *Lyve1-Cre:TdTomato* system (Pham et al., 2009). TdTomato+ cells were localized in visceral yolk sac and allantoic mesenchyme at E10.5 and in all labyrinthine ECs to E14.5 (Supplementary Figures S4A,B,D,F). Yolk sac hemogenic endothelia have been previously reported as *Lyve1+* (Lee et al., 2015). The TdTomato system has been known to over-report expression in highly expressed genes, so we therefore decided to compare our reporter with antibody/protein localization. Antibody staining partially matched the *Lyve1* reporter, indicating blood vessel ECs at E13.5 (Supplementary Figure S4C), but were absent in ECs at E10.5 and E15.5 (Supplementary Figures S4A,E). Antibody staining at E15.5 showed sporadic cells in the labyrinth and junctional zone, most likely to be macrophages. We conclude that *Lyve1+* is a pan-ECs marker in the placenta.

PROX1 is expressed in a subset of ECs in the cardinal vein which goes on to form lymphatics (Wigle et al., 2002). In doing so, it is a key initiator and marker of lymphatic ECs. However, there was no PROX1 expressed in placentas at E15.5 (data not shown). Given that there is no PROX1 expression in placental ECs and that LYVE1 and PDPN are not specific to a subset of ECs, this suggests that the placenta does not have a lymphatic network. However, these expression profiles suggest that the placenta may co-opt these lymphatic proteins to help make the placenta EC network. Whether the expression of these proteins make placental ECs “unique” or give them additional capabilities is yet to be explored.

### Maternal Iron Deficiency Impacts Placental Formation

Having now defined markers of placental arterial and venous ECs, we wanted to apply them to a disease model to see how impairing the embryonic cardiovascular system can affect their formation. As the placenta and heart develop in parallel, it is important to determine whether heart defects are primary or secondary to placental defects. We have recently shown that maternal ID causes congenital heart defects and sub-cutaneous oedema at E15.5 (Supplementary Figure S1A, Supplementary Table S1). Considering these cardiovascular defects, it is unknown how they may impact placental growth, or vice versa.

**FIGURE 2:**
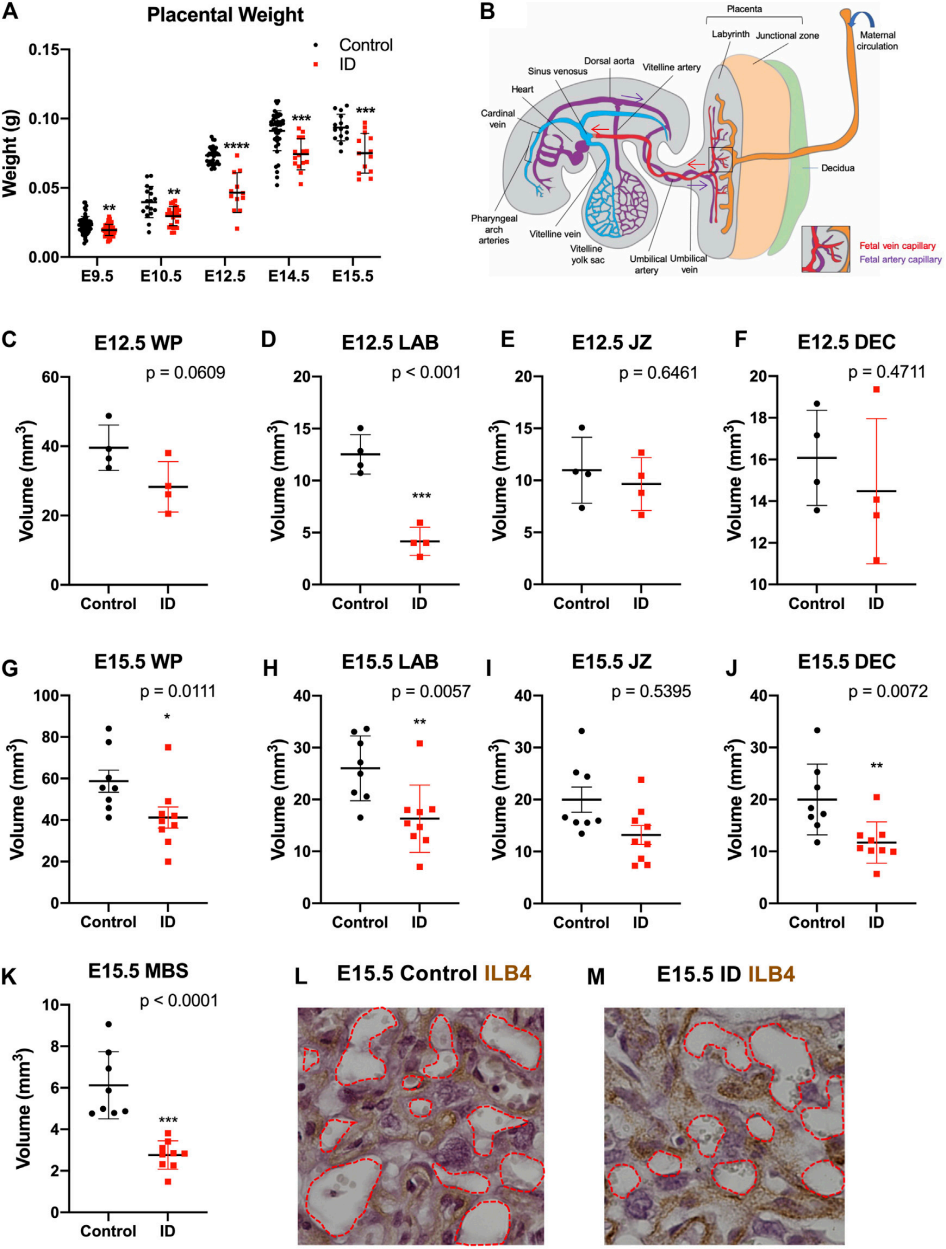
Maternal iron deficiency impacts placental formation, particularly to the labyrinth vasculature**. (A)** Placental wet weights from E9.5 to E15.5. After ID, placentas weighed less, most significantly at E12.5 (onset of embryonic death). Placental weights remained reduced to E15.5. **(B)** Diagrammatic representation of placenta-fetal blood flow in the mouse in mid-gestation with maternal blood spaces (orange), and fetal capillaries made up of PA (purple) and PV (red). Arrows show direction of blood flow. **(C–F)** Gross morphological volumes of placental compartments at E12.5 for whole placenta [WP, **(C)**], labyrinth [LAB, **(D)**], junctional zone [JZ, **(E)**], and decidua [DEC, **(F)**]. Labyrinth volume only was reduced after ID. **(G–L)** Gross morphological volumes of placental compartments at E15.5. Whole placental volume was reduced **(G)**, including compartments for the labyrinth **(H)** and decidua **(J)**. Maternal blood spaces [MBS, **(K)**] were quantified as Isolectin B4 (ILB4) negative staining and were reduced at E15.5. ILB4 staining of E15.5 placentas for control **(L)** and ID **(M)**, with red dashed line demarcating MBS. **(A)** Control: E9.5, *n* = 58; E10.5, *n* = 17; E12.5, *n* = 32; E14.5, *n* = 40; E15.5, *n* = 15. ID: E9.5, *n* = 59; E10.5, *n* = 24; E12.5, *n* = 12; E14.5, *n* = 15; E15.5, *n* = 13. (C–F) Control, *n* = 4; ID, *n* = 4. (G–K) Control, *n* = 8; ID, *n* = 9. Control (black dots) and ID (red squares). Data represented as mean ± SD. Data were analyzed by unpaired *t*-test unless otherwise stated. Mann–Whitney test was performed in A (E10.5 only), G–I. *p < 0.05, **p < 0.01, ***p < 0.001, and ****p < 0.0001. ID, iron deficient.

We first compared placental weight between ID and controls from E9.5 to E15.5 (Figure 2A). These were reduced between E9.5 and E15.5 as a result of ID. This was most significant at E12.5 (*p* < 0.0001), coincident with the beginning of embryonic death in approximately half of embryos (Kalisch Smith et al., 2021). The cause of this lethality is yet to be fully explored and therefore could be due to placental insufficiency (Navankasattusas et al., 2008). Placental weights of surviving embryos remained reduced at E14.5 and E15.5 (*p* < 0.01), albeit to a lesser extent. This could be due to survivor effects, possible because nutrients are reabsorbed from dead embryos. Gross placental volumes were assessed at E9.5, E12.5, and E15.5. Firstly, E9.5 placental tissue from somite-matched conceptuses was assessed by HREM to quantify total placental volume and volume of its compartments: decidua, the layer of parietal trophoblast giant cells (P-TGCs), ectoplacental cone (EPC), chorion, and allantois (Supplementary Figure S5). No significant changes were found in any placental compartment. To understand the disparity between these results and the reduced placental weight at this gestational age, we investigated somite number at E9.5. ID caused a reduction in somite number (*p* < 0.001, Supplementary Figure S5J, control = 22, ID = 20 somites). This suggests that ID embryos and placentas have a slight developmental delay, but this does not affect relative placental size.

**FIGURE 3:**
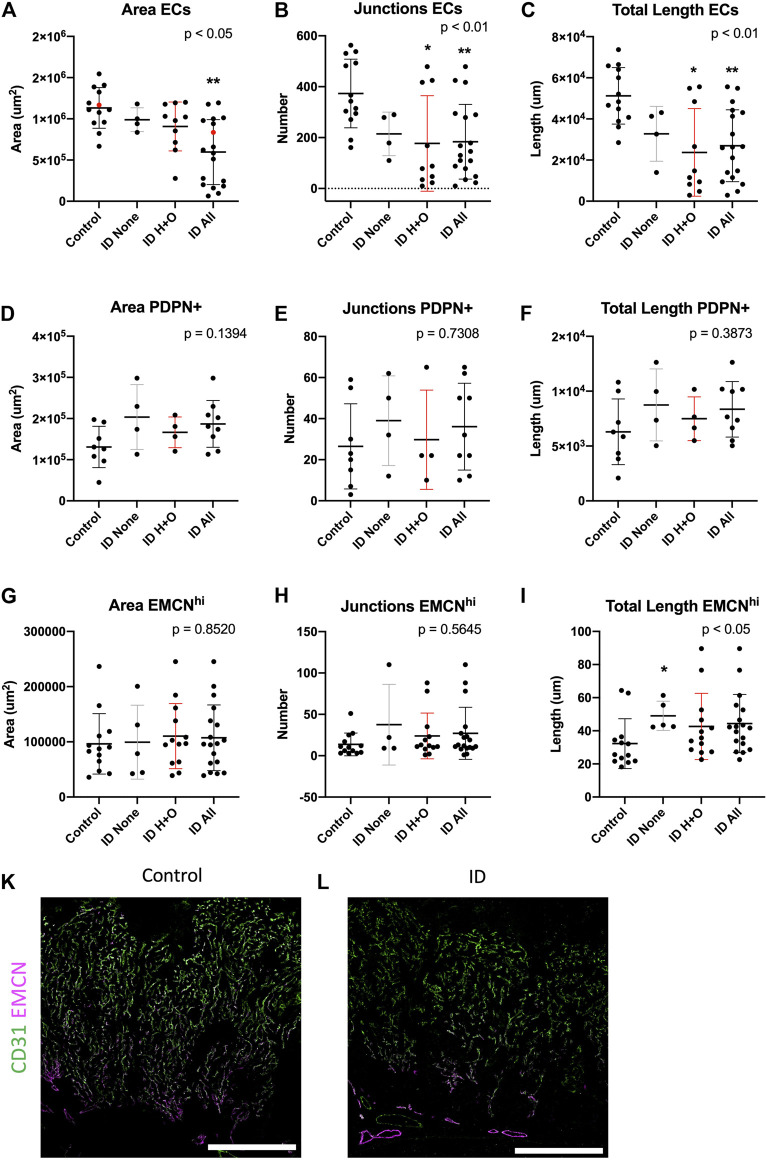
Maternal iron deficiency reduces total but not venous-specific ECs in the E15.5 placenta. Total ECs **(A–C)**, PDPN+ ECs **(D–F)**, and veins (EMCN^hi^) **(G–I)** were assessed for vessel area, number of junctions, and total length. ID samples were stratified for those with heart defects and oedema (ID H + O) or those with none (ID none). Red points in (A) correspond to placental images in (K, control) and (L, ID), showing CD31 (pan-EC) and thresholded EMCN to highlight strong venous expression. IHC was performed for CD31 and EMCN. Data represented as mean ± SD. Data were analyzed by ordinary one-way ANOVA if normally distributed **(D–F)** or, if not, by Kruskal–Wallis test **(A–C,G–I)**. Post-hoc analysis was compared to the control group; *p < 0.05 and **p < 0.01. Control, n = 13 **(A–C, G–I)** and 8 **(D–F)**. ID, n = 18 (A–C, G–I) and 9 **(D–F)**. ID, iron deficient. Scale bars (white) = 200 um.

We assessed later placental stages after ID using unbiased stereology. At E12.5 after ID, we found that, while the whole placental trended toward a decrease in volume (*p* = 0.0609, Figure 2C), the labyrinth vascular compartment was significantly reduced (*p* < 0.001, Figure 2D). The junctional zone and decidua remained unchanged (Figures 2E,F, see Figure 3B for diagrammatic representation of placental zones). By E15.5, quantification showed that ID reduced volumes of the whole placenta (*p* = 0.011, Figure 2G), labyrinth (*p* = 0.0057, Figure 2H), and decidua (*p* = 0.0057, Figure 2J). No change was found in the junctional zone compartment (Figure 2I). Embryonic capillaries (blood spaces) were identified using the marker ILB4 (Figures 2L,M). Maternal blood space was markedly reduced after ID (*p* < 0.0001, Figure 2K). Maternal blood spaces were identified by an absence of staining, and the presence of large nuclei of sinusoidal trophoblast giant cells lining these blood pools.

### Maternal ID Impacts Placental Arterial but Not Venous Networks

We next sought to assess placental arterial and venous vasculature after ID, stratifying for those from embryos with and without heart defects and oedema (H + O), see Supplementary Table S1 and Supplementary Figure S6 for summary of heart defects, HREM image stacks for representative control (Movie1) or ID (Movie2). Despite these defects, ID did not impact ventricular volume (Supplementary Figure S6A). Midline cross-sections of placentas were estimated for area occupied by placental blood vessels. Midline sections were stained for EMCN and counted for EMCN high (venous ECs) and total ECs (CD31^+^). ID caused a reduction in the total area, number of junctions, and total length of CD31 ^+^ vessels [Figures 3A–C; see representative images for control (Fig.4K) and ID placentas (Fig.3L)]. Post-hoc analysis revealed this effect in Fig. 4B and C was driven by ID H + O embryos (*p* < 0.05 vs. control) and not by ID embryos with none. ID H + O placentas also showed greater variation than ID embryos with no defects. PDPN+ blood vessels were also assessed but were unchanged with area, junctions, or length (Figures 3D–F). Finally, EMCN high venous vessels were assessed for area, junctions, and total length after ID or ID H + O but found no differences (Figures 3G–I). Surprisingly, ID embryos with no defects increased the total length of EMCN-high vessels compared with controls (Figure 3I). Because ID reduced total EC vessels without changing venous vessels, this suggests that ID impacts primarily growth of the placental arterial tree. The placental arteries receive high pressure blood pumped from the heart to the remaining embryo. Therefore, heart defects causing reduced blood flow to placenta are likely to affect arterial expansion.

Furthermore, no changes were found between control and ID of CD31+ blood vessels at E12.5, assessed for area, number of junctions, and total length (Supplementary Figures S7A–C). Given that heart defects were shown by this stage (Supplementary Figure S1A), this suggests that physiological changes in the heart initiate secondary placental vascular defects.

## Discussion

We have found that the placental vasculature has some similarities but many differences from the embryonic vasculature and is thus a potentially unique endothelial organ bed. The differences in the expression patterns of between embryonic and placental ECs may be representative of their distinctive developmental origins or indicate that they can respond to different local cues. In the placenta, the interhemal membrane is the site of nutrient and waste exchange between the maternal and embryonic circulatory systems. It consists of three layers of cells, with the placental EC, lining the embryonic vasculature, juxtaposed to two layers of syncytiotrophoblasts, lining the maternal blood spaces. Thus, the unique molecular patterning of the placental EC could result in response to signals from the syncytiotrophoblasts. Alternatively, placental ECs may differentiate differently to embryonic ECs due to differences in blood flow rate, vascular resistance, or oxygenation between the placenta and embryo. For example, placental arterioles carry deoxygenated blood, returning from the embryo to the placenta, whereas embryonic arterioles carry oxygenated blood. The differences that we have identified in the molecular patterning of the placental vasculature may influence how placental ECs respond to different perturbations, be that extrinsic (e.g., maternal nutrition) or intrinsic (genomic mutations). Further research is now warranted to classify and compare placental and embryonic ECs, such as by in-depth computational and *in situ* approaches. These differences are important to understand how placental ECs develop, function, and interact with adjacent cell types.

Previous animal models investigating the impacts of maternal ID on placental form and function have focused analyses at late gestation. However, the results are inconsistent between different models. For example, in the rat placental and labyrinthine, volume is increased at GD21 (Lewis et al., 2001) and placental weight to body weight ratio is increased at GD21 (Woodman et al., 2017). However, in ID mice, there was no change in placental weight by E15.5 (Sangkhae et al., 2020). These are less severe phenotypes compared with our results and may be due to the different induction times and durations to create ID. The above three models were induced 1–2 weeks prior to mating, while we induced from weaning. Conversely, these results could indicate compensatory increases in placental vascularization of surviving embryos to increase oxygen supply.

Evidence that a link between congenital heart defects (CHDs) and placental perturbations in humans is emerging. In a study of 924,422 live-born Danish babies, they showed that CHDs including teratology of Fallot, DORV, and VSD were associated with smaller placental size at birth (Matthiesen et al., 2016). Additional CHDs including hypoplastic left heart syndrome have been associated with placental vascular defects (Jones et al., 2015; Rychik et al., 2018; Courtney et al., 2020). We point that, in our ID model, the placental defects are secondary to the heart defects. The evidence for this is two-fold. Firstly, maternal ID does not change the area of placental blood vessels until E15.5, whereas the heart defects are already detectable at E10.5 (Kalisch Smith et al., 2021). In addition, ID embryos without heart defects showed no placental defects. Secondly, in addition to the heart defects, ID embryos at E12.5 have delayed remodeling of the pharyngeal arch arteries. Taken together, these abnormalities may perturb and reduce blood flow into the placenta. Other models restricting blood flow to the placenta that result in perturbed labyrinth formation are often associated with heart defects. These include knockout of *Ncx1*, a cardiac Na^+^/Ca^2+^ exchanger, which impacts embryonic heart rate (Cho et al., 2003) and therefore blood flow. In addition, administration of excess glucocorticoids has also shown reduced placental vascularization that coincides with delayed heart development (Wyrwoll et al., 2016). Many genetic knockout models show heart and placental defects such *Cxadr* (Outhwaite et al., 2019), *Fltr2* (Tai-Nagara et al., 2017), *Hoxa13* (Shaut et al., 2008), and p38a MAPK (Adams et al., 2000) (reviewed by Maslen, 2018; Camm et al., 2018).

Adaptations of the placental arterial tree in response to genetic or environmental challenge are commonplace. Other models including hypomorphic *Gcm1* expression (Bainbridge et al., 2012) and exposure of mouse dams to cigarette smoke (polycyclic aromatic hydrocarbons) (Detmar et al., 2008), both increased the placental arterial tree. Knockout of *Cited2*, however, affected both arterial and venous trees (Withington et al., 2006). An *Unc5b* (netrin receptor) knockout showed that a reduction in placental arteriole formation was associated with blood flow reversal to the embryo and was the cause of embryonic lethality at E13 (Navankasattusas et al., 2008). This could similarly occur after ID, where we see lethality beginning by E12.5 (Kalisch Smith et al., 2021). By contrast, it is currently unknown what teratogens, if any, might impact primarily the venous placental vasculature.

Blood flow and shear stress from the heart can have a big impact on vascular growth and development. Fluid shear stress is the frictional force caused by blood flow acting on ECs. It is a mechanical cue required for EC proliferation and remodeling and upregulates several different transcriptional pathways (Hahn and Schwartz, 2009). In the embryo, Notch1 expression increases from the onset of flow (Jones et al., 2015), whereas important flow-induced transcription factors include KLF2 (Kruppel-like factor 2) and eNOS (endothelial nitric oxide synthase) (Hahn and Schwartz, 2009; Ku et al., 2019). A previous study, which reduced shear stress by lowering embryonic hematocrit levels, inhibited remodeling of the yolk sac vascular plexus (Lucitti et al., 2007). Hemodynamic forces are necessary for vascular expansion and development of the placenta as well. While the impacts of shear stress in the placenta are currently lacking in animal models, computational modeling of placental capillary shear stress in the context of fetal growth restriction can affect vascular branching (Tun et al., 2019).

## Conclusion

We show that the placenta is a potentially unique endothelial bed, expressing only a subset of classical embryonic EC markers. Further research is now warranted to compare placental ECs to other unique organ beds in the embryo, such as the heart and lung. While true placental venous markers remain elusive, we have shown that venous ECs/venules express higher EMCN than their arterial counterparts. These markers can be used to better understand placental development and dysfunction. Lastly, we have investigated one common maternal condition‐ maternal iron deficiency and shown an arterial-specific defect in, placenta.

## Data Availability

The original contributions presented in the study are included in the article/Supplementary Material, further inquiries can be directed to the corresponding authors.
