# Supplementary material for: Analysis of Placental Arteriovenous Formation Reveals New Insights Into Embryos With Congenital Heart Defects
Source: Front Genet. 2022 Jan 19;12:806136. doi: 10.3389/fgene.2021.806136 (PMC8809359; doi:10.3389/fgene.2021.806136)
Supplement: Supplementary file 2 [file DataSheet1.pdf]

Supplementary Figures

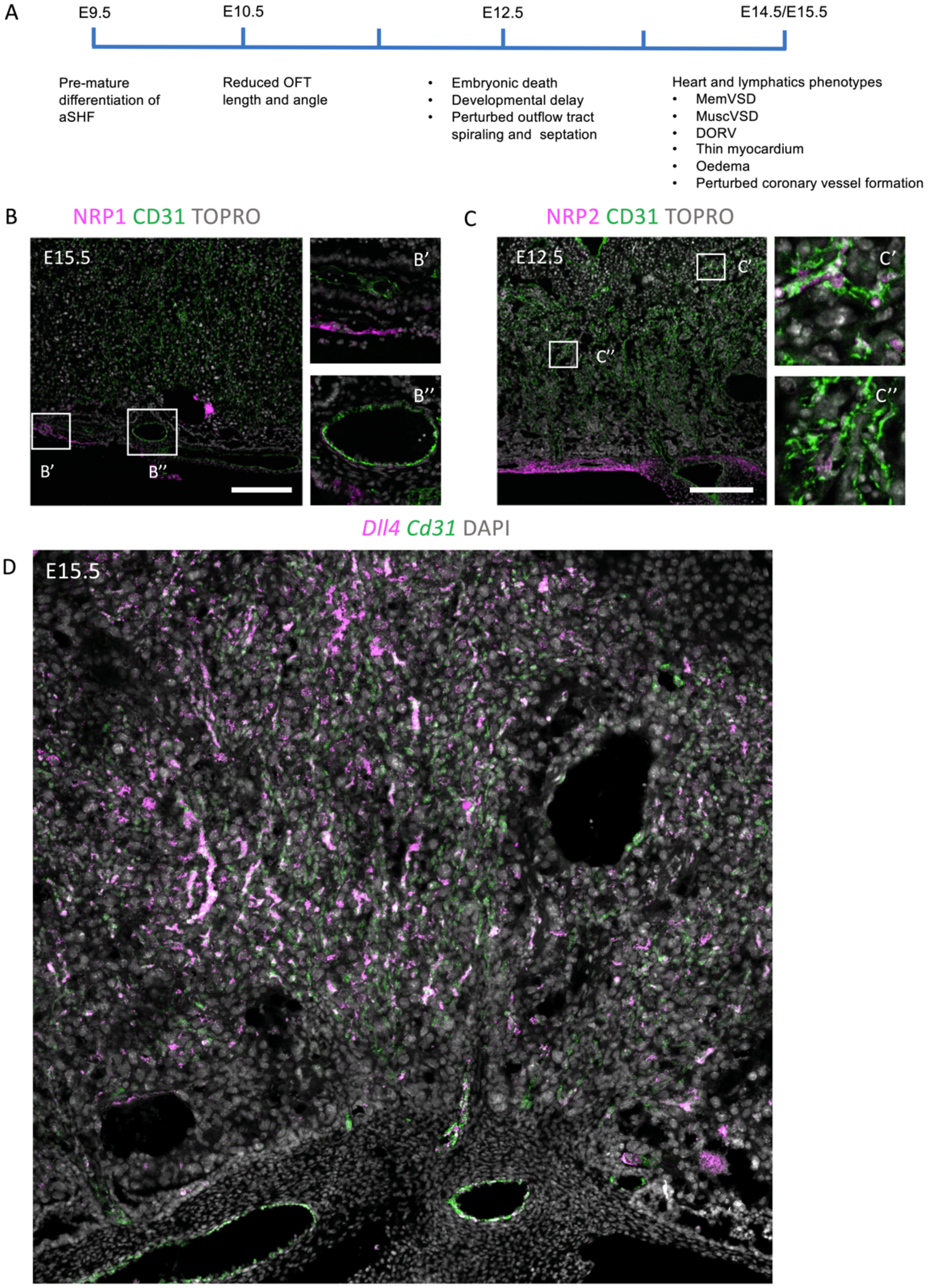

Supplemental Figure 1. (A) Summary of heart defects after maternal iron deficiency assessed from E9.5 to E15.5. **(B)** NRP1 expression at E15.5. **(C)** NRP2 staining at E12.5**.** White boxes show magnified views from B-C (‘). **(D)** Dll4 RNAscope staining, magnified version of Fig.1D. Scale bars (white) in B and C = 200um. aSHF; anterior second heart field, DORV; double outlet right ventricle, OFT; outflow tract, MemVSD; membranous ventricular septal defect, MuscVSD; muscular ventricular septal defect.

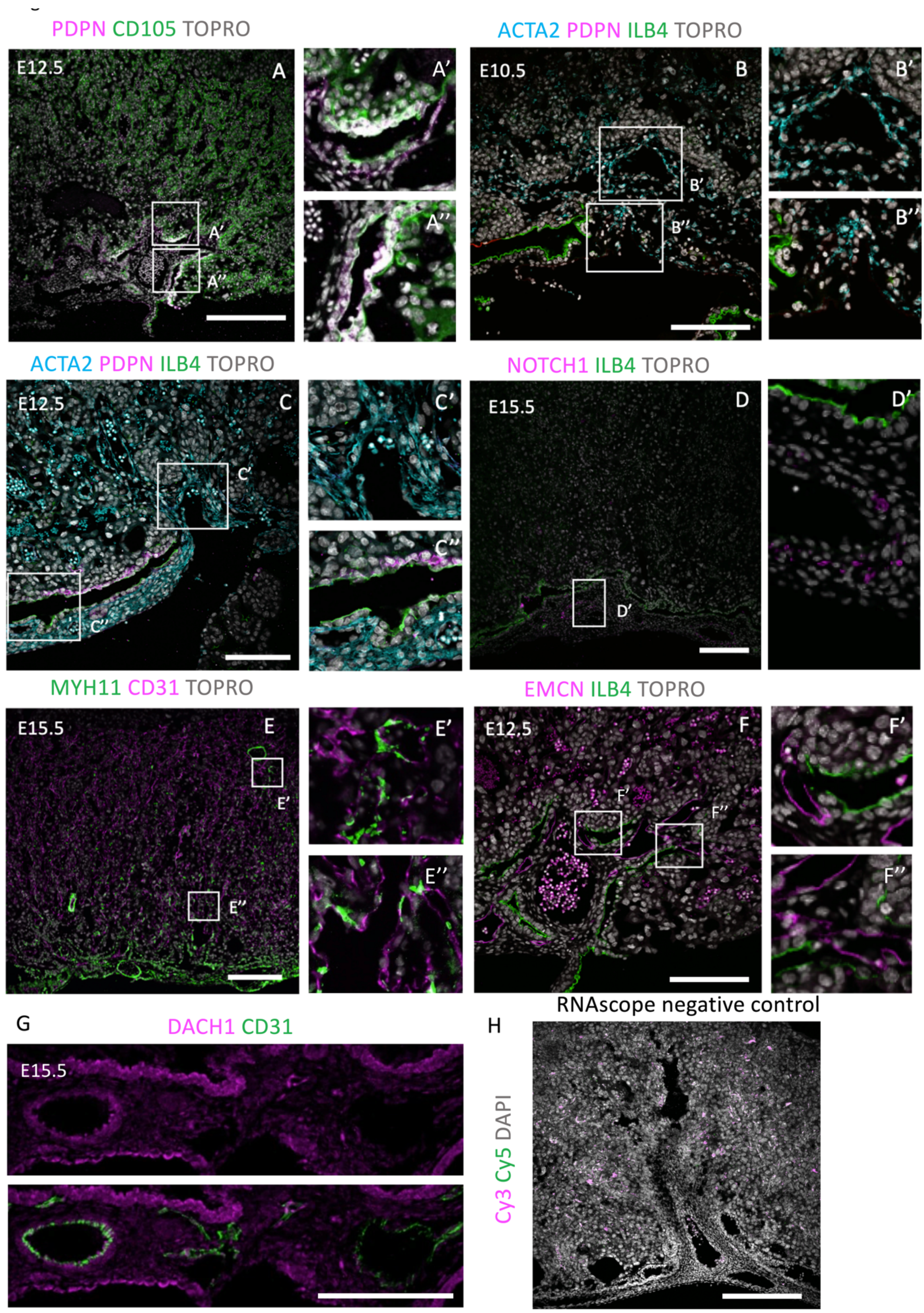

Supplemental Figure 2. Protein localisation of common embryonic vascular-associated genes from E10.5 to E15.5. Figures from A-G show IHC, while (H) shows RNAscope. White boxes show magnified views from A-F ('). PDPN; podoplanin. CD105

(endoglin). ILB4; Isolectin B4. ASMA; Alpha smooth muscle actin. SM-MHC; Smooth muscle myosin heavy chain. EMCN; Endomucin. Scale bars (white) = 200um.

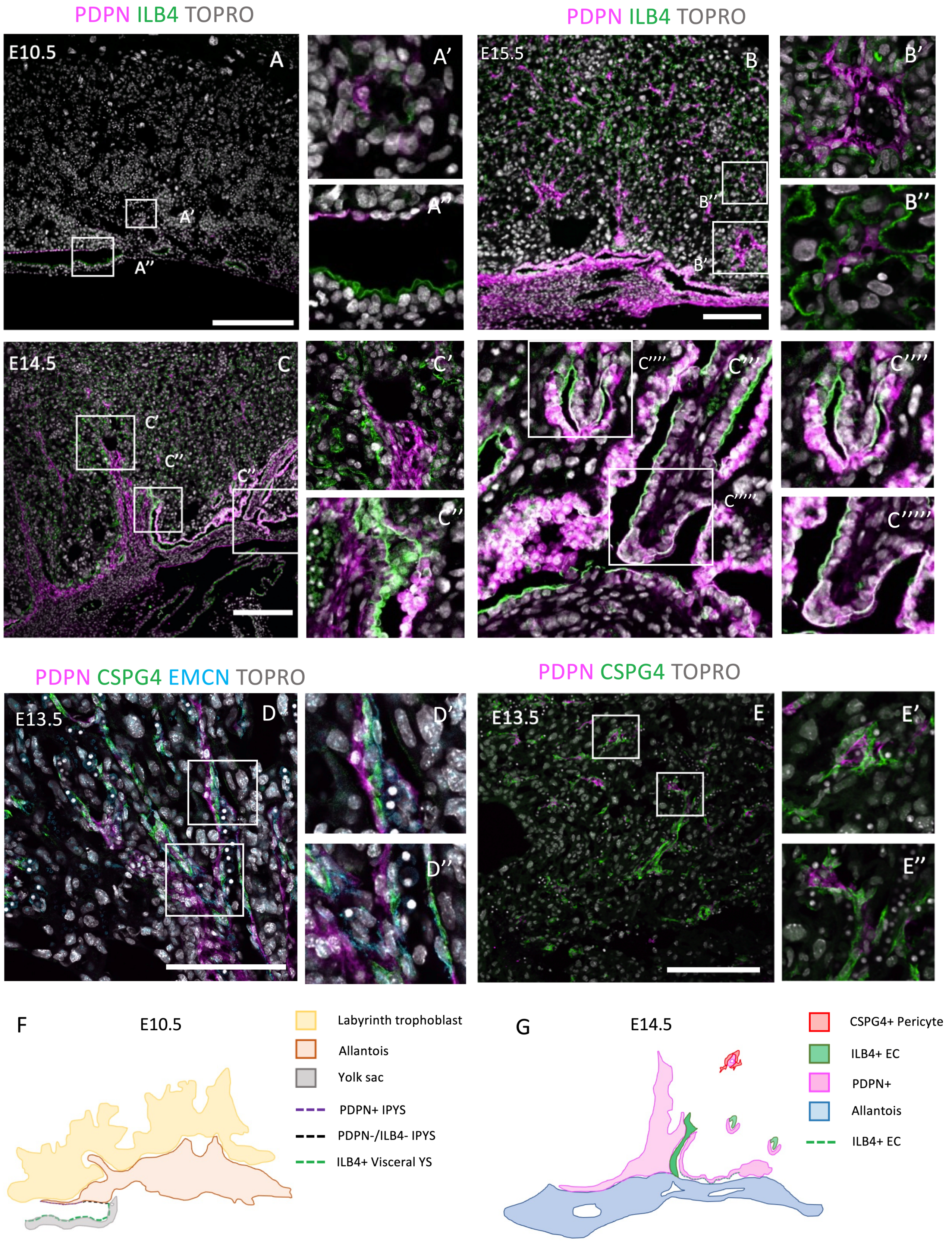

Supplemental Figure 3. Investigation of common embryonic lymphatic genes during placentation. Podoplanin (PDPN, A-E) was investigated from E10.5 to E15.5 in combination with endothelial markers ILB4 and CD31, venous marker (EMCN high, arterial EMCN low), and pericyte marker CSPG4 (NG2, chondroitin sulphate proteoglycan). IHC

was performed for all figures in A-E. The boxes outline the areas shown at higher magnification in A-E (‘). Diagrammatic representations of PDPN expression at E10.5 (F) and E14.5 (G). Scale bars (white) = 200um.

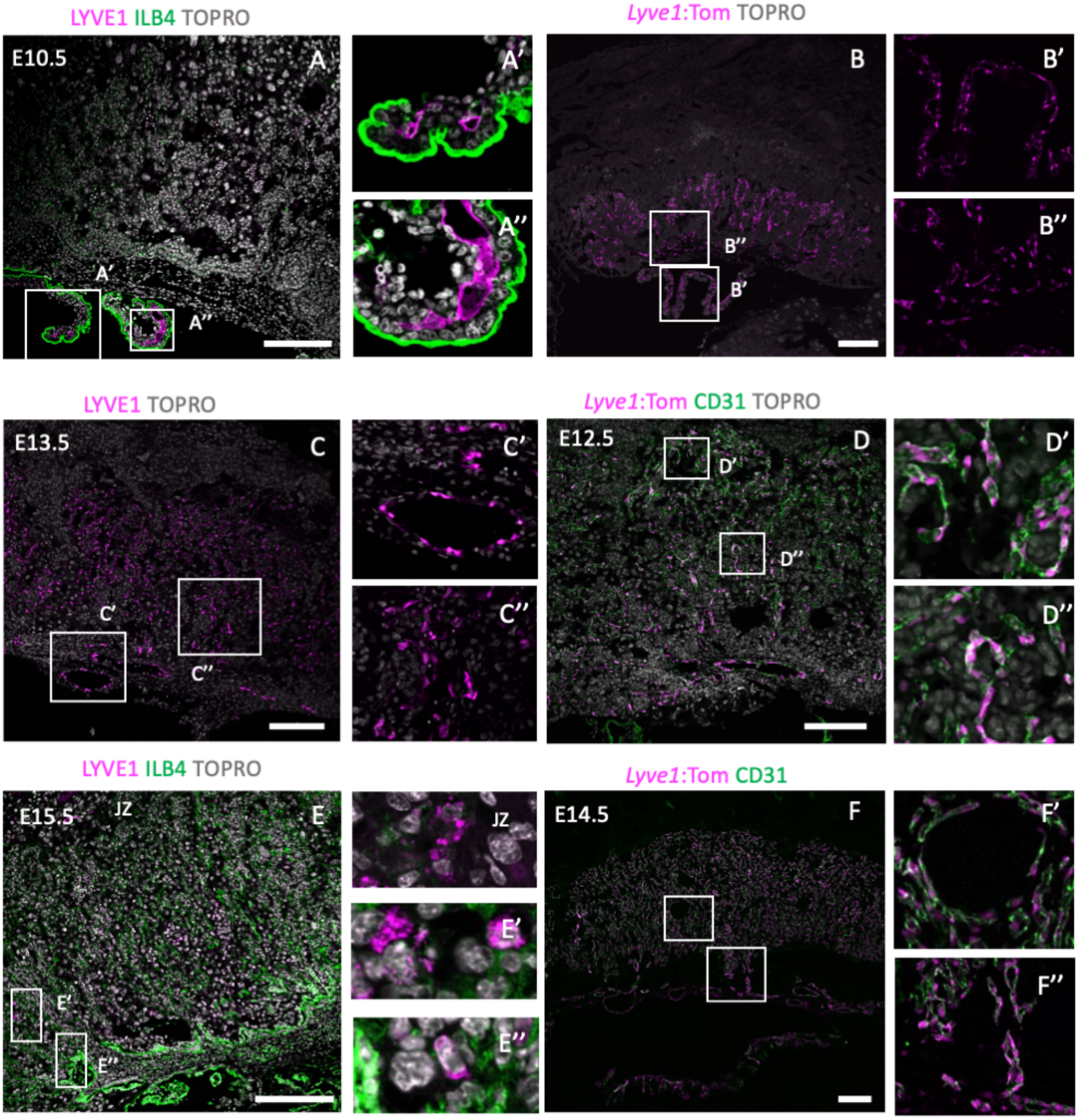

Supplemental Figure 4. *Lyve1* expression during labyrinth development. Comparative staining for LYVE1 antibody (IHC, A, C, E) versus Lyve1-Cre:Tomato staining (B, D, F). JZ; junctional zone. ILB4; Isolectin B4. White boxes show magnified views in A-F (‘). Scale bars (white) = 200um.

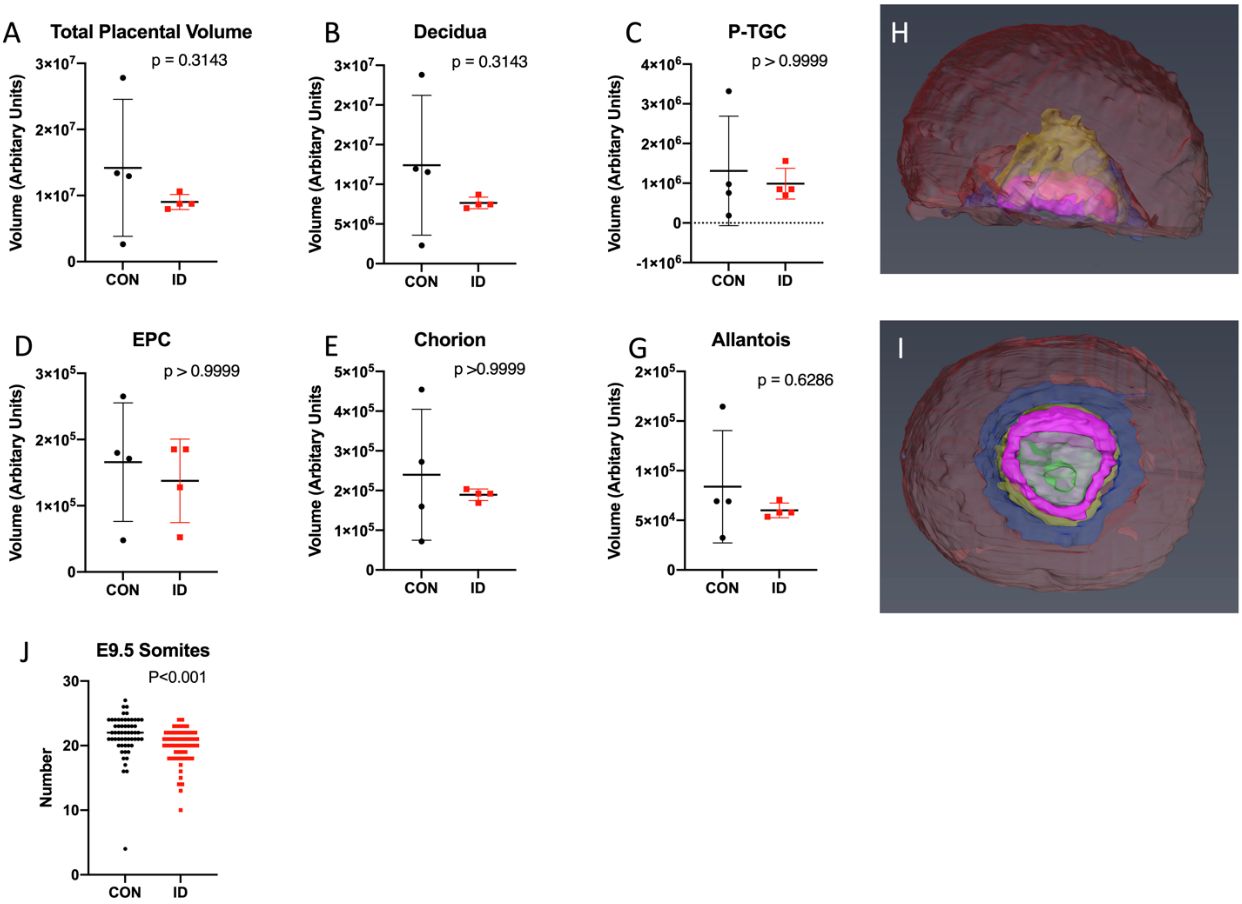

Supplemental Figure 5. Maternal iron deficiency does not alter placental morphology at E9.5. Whole placental tissue from somite matched embryos was sectioned and imaged by HREM. 3D reconstructions were made using Amira software, and rendered for decidua (B, red), parietal trophoblast giant cells (P-TGC, C, blue), ectoplacental cone (EPC, D, yellow), chorion (E, pink), and allantois (G, green). No significant differences were found for any compartment (B-G), or for all compartments combined (A). (J) Somite number of E9.5 embryos. (A-G) Control (black circles): n=4, ID (red squares): n=4. Data represented as mean ± SD. (J) Control n=57, ID n=58, data represented as median ± 95% confidence interval. All data was analysed by Mann-Whitney tests. ID; Iron Deficient.

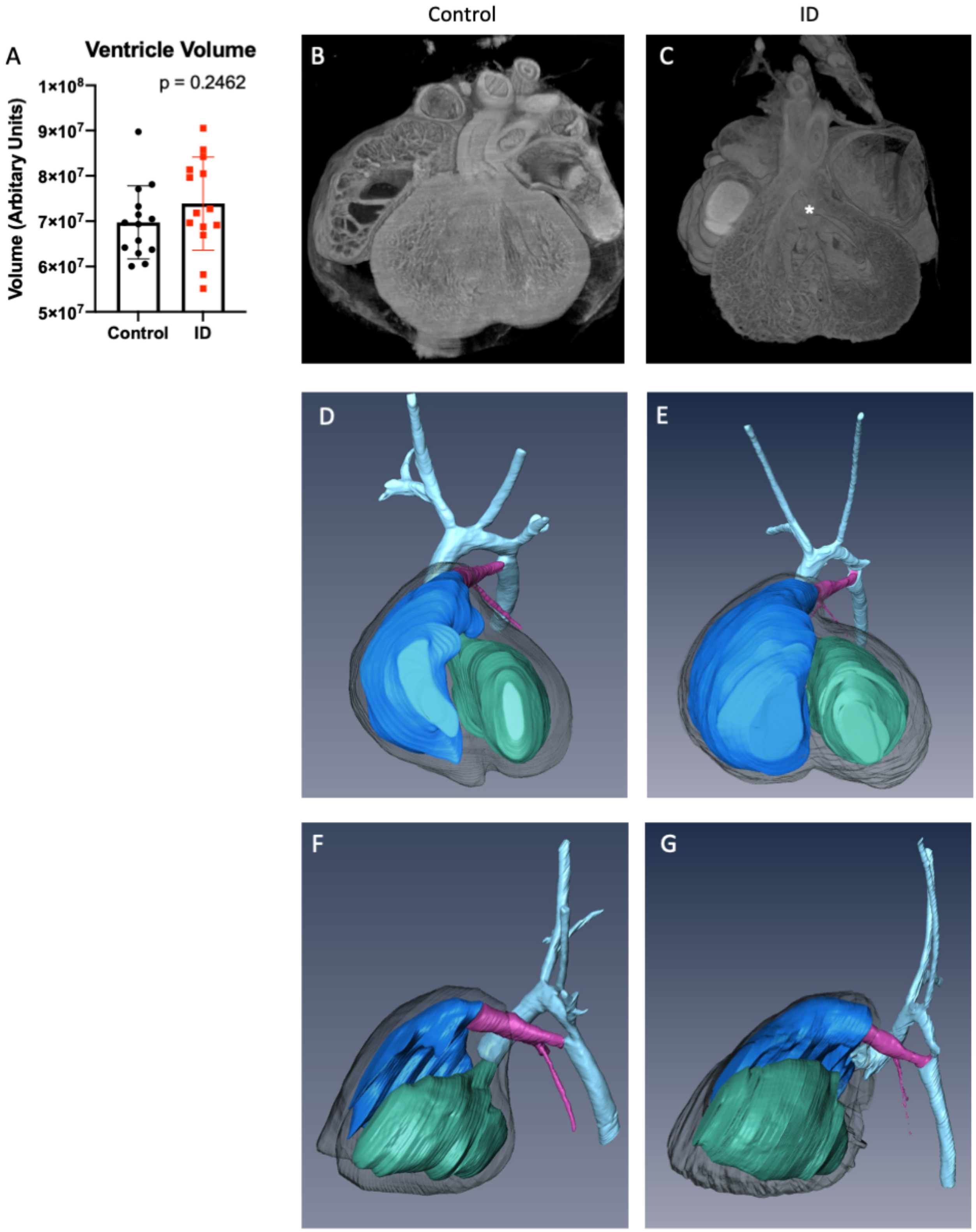

Supplemental Figure 6. Maternal ID does not impact ventricular volume at E15.5 but does cause congenital heart defects. (A) Heart ventricular volume at E15.5. (B) Control and (C) Iron Deficient (ID) 3D reconstructions of E15.5 hearts. Asterix in (C) denotes membranous VSD. This heart also shows a thin myocardium. (D,F) Control and (E,G) ID Amira models of E15.5 hearts. Control shows a normal aortic arch while the ID has a double-outlet right ventricle.

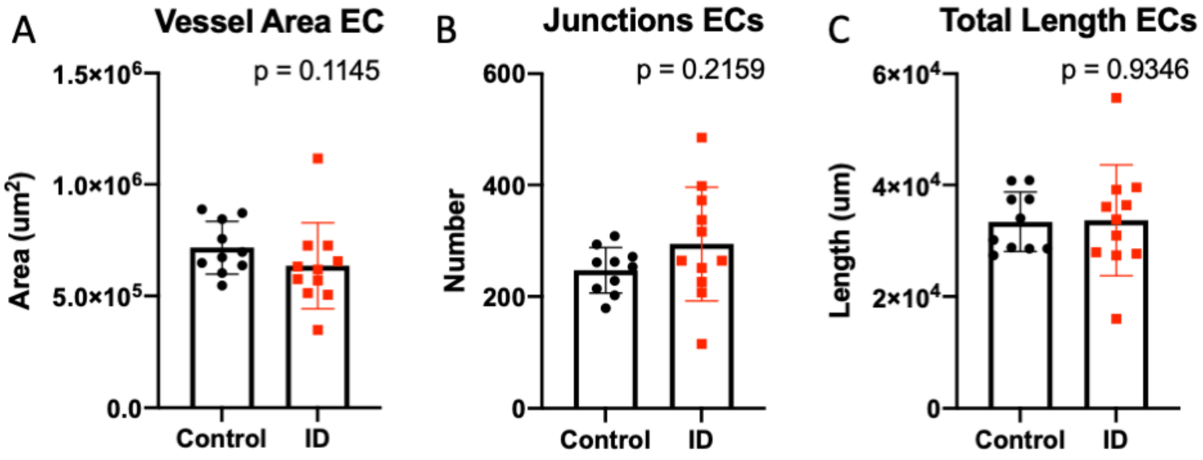

Supplemental Figure 7. Maternal ID does not alter E12.5 placental blood vessels. Vessel area (A), number of junctions (B), and total length (C) of ECs were quantified. Data represented as mean ± SD. Data was analysed by Mann-Whitney tests (A,B) or unpaired t tests (C). Control n= 10, ID n=11. ID; Iron Deficient.

Cardiovascular organ	Type of defect	Control	ID	ID (original cohort)
**Single heart defects**	ASD	0/16	1/16	0/42
	memVSD	0/16	1/16	5/42
	muscVSD	0/16	0/16	11/42
**Multiple heart defects**	memVSD + muscVSD	0/16	3/16	4/42
	memVSD + AVSD	0/16	0/16	0/42
	muscVSD + AVSD	0/16	0/16	4/42
	OFT + memVSD	0/16	0/16	6/42
	OFT + memVSD + muscVSD + AVSD	0/16	4/16	4/42
	OFT + muscVSD + AVSD	0/16	0/16	1/42
	memVSD + AVSD	0/16	1/16	0/42
**Total normal**		16	6/16	12/42
**Total abnormal**		0	10/16 (63%)	30/42 (71%)
**Aortic arch defects**	IAA	0/16	1/16	3/40
	A-RSA	0/16	016	1/40
	R-LCC	0/16	0/16	1/40
	Right sided-AA	0/16	1/16	1/40
	Aortic stenosis	0/16	2/16	-
**Total normal**		16	6/16	35/40
**Total abnormal**		0	3/16 (18%)	5/40 (13%)
**Lymphatic defects**	Oedema	0/16	8/16 (50%)	22/42 (52%)

Table S1. New cohort of maternal ID embryos at E15.5 for correlation analyses of placental and cardiovascular phenotypes. A-RSA; aberrant right subclavian artery, ASD; atrial septal defect, AVSD; atrioventricular septal defect, IAA; interrupted aortic arch, OFT; outflow tract defects including double-outlet right ventricle and overriding aorta, memVSD; membranous ventricular septal defect, muscVSD; muscular ventricular septal defect, R-LCC; retroesophageal left subclavian artery. Note: An embryo may have more than one phenotype.

Supplemental Movie 1. HREM image stacks for representative control heart at E15.5.

Supplemental Movie 2. HREM image stacks for representative iron deficient heart at E15.5.
